# Cushing Disease After Treatment of Nonfunctional Pituitary Adenoma: A Case Report and Literature Review

**DOI:** 10.1097/MD.0000000000002134

**Published:** 2015-12-28

**Authors:** Hongjuan Fang, Rui Tian, Huanwen Wu, Jian Xu, Hong Fan, Jian Zhou, Liyong Zhong

**Affiliations:** From the Department of Endocrinology, Beijing Tiantan Hospital, Capital Medical University (HF, JX, HF, LZ), Department of Neurosurgery (RT), Department of Pathology, Peking Union Medical College Hospital, Peking Union Medical College, Chinese Academy of Medical Sciences (CAMS) (HW); and Department of Radiology, Beijing Tiantan Hospital, Capital Medical University, Beijing, China (JZ).

## Abstract

We describe a very rare case of nonfunctional pituitary adenoma (NFPA) that exhibited corticotrophic activity after resection and radiotherapy. The possible mechanisms of the transformation from NFPA to Cushing disease (CD) are discussed.

A 43-year-old man presented with impaired vision, bilateral frontal headaches, and hyposexuality. He had no symptoms suggestive of hypercortisolism, and 8 am plasma cortisol concentration was 67.88 ng/mL. Brain imaging revealed a 15 × 15 × 21-mm sellar mass suggestive of a macroadenoma. The tumor was resected by transsphenoidal surgery and identified by immunohistochemical analysis as a chromophobic adenoma that did not stain for pituitary hormones. The patient was treated with prednisone and levothyroxine replacement therapy. After a third recurrence, the patient presented with clinical features and physical signs of Cushing syndrome. Plasma adrenocorticotropic hormone (ACTH) and cortisol concentrations were elevated, and there was a loss of circadian rhythms. Inferior petrosal sinus sampling after desmopressin showed the central–peripheral ACTH ratio was greater than 3:1. A repeat transsphenoidal resection was undertaken. Immunohistochemistry revealed ACTH positivity. Three months following surgery, imaging showed little residual tumor, but plasma ACTH remained elevated. He was referred for postoperative Gamma Knife radiotherapy.

The immunological activity and biological features of the hormones secreted from a pituitary adenoma vary with time. Because long-term outcomes are unpredictable, postoperative follow-up is essential to detect postoperative transformation from NFPA to CD.

## INTRODUCTION

Adrenocorticotropic hormone (ACTH)-secreting pituitary tumors, which cause Cushing disease (CD), account for 5% to 10% of pituitary adenomas. The incidence of CD is reported to vary between 2.4 per million persons in Europe and 8 per million in those <65 years of age in the United States.^1,2^ Pituitary adenomas can be classified by their pathologic or functional characteristics, typical or atypical clinical behavior, and whether they are invasive or noninvasive, and aggressive or nonaggressive.^3^ Functional classification is based on a tumor's endocrine activity as determined by serum hormone concentrations and pituitary tissue cellular hormone secretion detected by immunohistochemical staining.^3^

Rarely, nonfunctional pituitary adenomas (NFPAs) may gain secretory function, but there have been a few case reports of metamorphosis to CD.^4–17^ We describe the clinical course of a man with a proven NFPA whose tumor became functional and began to secrete biologically active ACTH that led to severe Cushing syndrome, review published reports of other cases, and discuss the putative mechanisms of tumor transformation.

The patient and his wife gave written informed consent for this publication.

## CASE REPORT

**FIGURE 1:**
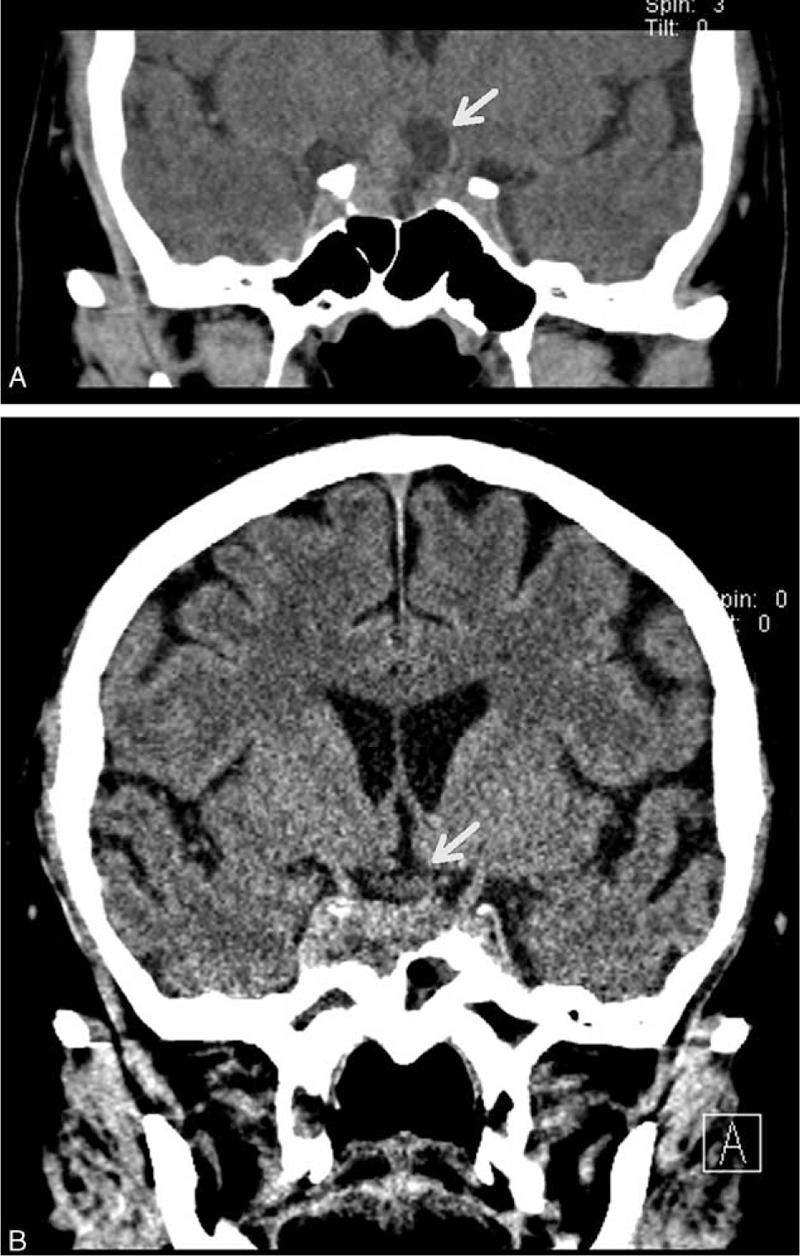
Head computed tomography (CT) scan: (A) before the 1st operation, coronal CT with multiple planar reconstruction shows a dilated sella turcica with a heterogeneously attenuated mass (white arrow); (B) at the 4th recurrence (October 2014), coronal CT multiplanar reconstruction shows the irregular suprasellar anatomy (white arrow) (likely a consequence of 3 previous operations and Gamma Knife radiotherapy).

**FIGURE 2:**
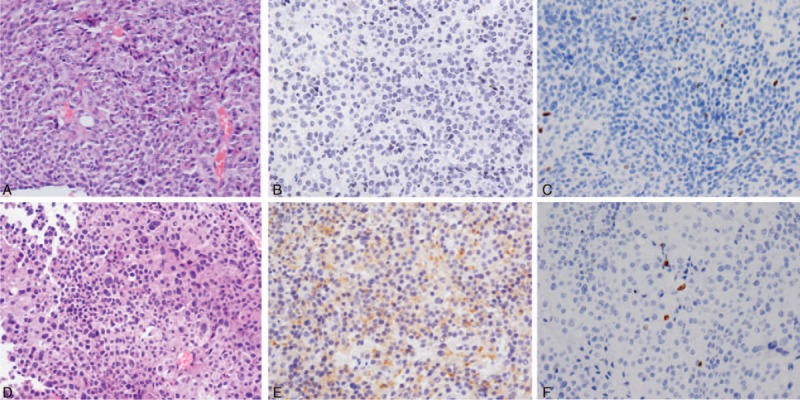
(A) Histopathology findings of tissue sampled during the first operation (HE staining) showing (B) no staining for ACTH and (C) a Ki-67 proliferation index of approximately 3% (magnification ×200): (D) histopathology findings of tissue sampled during the 4th operation (HE staining) showing (E) strong staining for ACTH, and (F) a Ki-67 proliferation index of approximately 2% (magnification ×200). ACTH = adrenocorticotropic hormone, HE = hematoxylin–eosin.

A 43-year-old man was referred in March 2008 with an 8-month history of progressive headache, loss of peripheral vision, and hyposexuality. He did not have polyuria, polydipsia, or any symptoms of hypercortisolism, hypermetabolism, or acromegaly. He was 1.66 m tall and weighed 76 kg (body mass index [BMI] of 27.6 kg/m^2^). Endocrine investigations were suggestive of hypotestosteronism, but plasma cortisol, prolactin, growth hormone, and thyroid hormone concentrations were normal (Table 1). Computed tomography (CT) showed enlargement of the sella turcica containing a well-defined 15 × 15 × 21-mm mass compressing the optic chiasm, infiltrating the right side of the cavernous sinus and extending around the right internal carotid artery (Figure 1A). The patient underwent transsphenoidal surgery, and the mass was partially resected. Conventional histopathological examination of the excised tissue showed tumor cells consistent with a pituitary chromophobic adenoma (Figure 2A-C), and stained negative for ACTH (Figure 2B) and other pituitary hormones (growth hormone, prolactin, luteinizing hormone, follicle stimulating hormone, and thyroid-stimulating hormone). The Ki-67 proliferative index was 3% (Figure 2C). Postoperative endocrine studies reported blood concentrations of total triiodothyronine of 0.84 nmol/L (normal range 1.18–2.23 nmol/L), total thyroxine of 70.22 nmol/L (normal range 57.92–154.44 nmol/L), thyroid-stimulating hormone of 0.292 μIU/mL (normal range 0.47–4.64 μIU/mL), and cortisol of 580.46 ng/mL (normal range 30.0–230.0 ng/mL). The latter was higher than the physiological upper limit, which was considered to be the result of surgical trauma and stress, and hydrocortisone therapy. Replacement therapy with prednisone 7.5 mg once daily and levothyroxine 50 μg once daily was begun postoperatively. Postoperative endocrine studies 3 months later found that the serum cortisol concentration was 5.60 μg/dL (normal range 5.0–25.0 μg/dL) at 8 am, the dose of prednisone decreased to 5 mg qd.

**TABLE 1:**
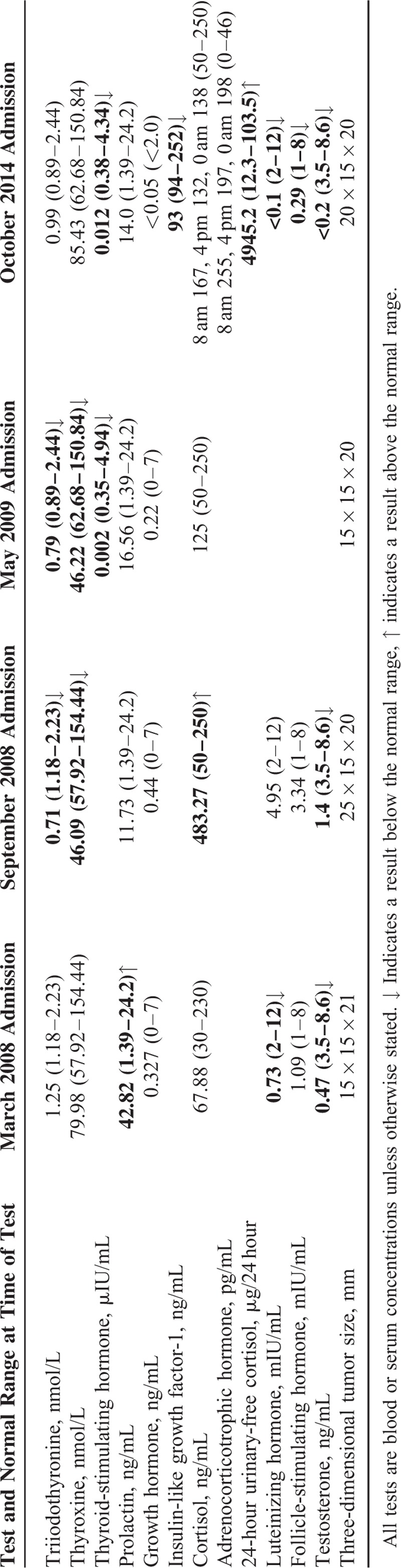
Endocrine Investigations and Tumor Size Overtime

Six months later, in September 2008, the patient presented with a 2-month history of headaches and progressive visual impairment. Follow-up magnetic resonance imaging showed substantial growth of the residual tumor into a mass measuring 25 × 15 × 20 mm. Although the patient reported no symptoms of hypercortisolism, preoperative endocrine studies revealed hypothyroidism, hypercortisolemia, and hypotestosteronism (Table 1). The levothyroxine dose was increased to 100 μg once daily, prednisone was maintained 5 mg once daily. A second transsphenoidal partial excision of the tumor was performed, and histopathologic analysis of the tissue revealed megakaryocytes and sporadic ACTH-positive cells with a Ki-67 proliferative index of 1.5%. There was no immunohistochemical evidence of other pituitary hormones, suggestive of a pituitary adenoma.

In June 2009 (15 months after the first surgery), the patient presented again with a 2-month history of headache and blurred vision. Imaging suggested a further recurrence of the adenoma. After the second partial resection, the patient checked his plasma cortisol regularly, and the prednisone dose was adjusted according to the cortisol levels and electrolyte concentration. And the prednisone dose was maintained 5 mg once daily. Preoperative endocrine testing suggested persistent central hypothyroidism, and the levothyroxine replacement dose was increased to 125 μg once daily (Table 1). The size and location of the tumor necessitated a subfrontal craniotomy, during which a nearly complete resection of the 15 × 15 × 20 mm tumor was achieved. Postoperative histopathologic examination yielded evidence of an actively growing pituitary adenoma, with a Ki-67 proliferative index of 1.5%. There was no evidence of staining for any of the adenohypophyseal hormones.

The number and frequency of recurrences called into question the benign nature of the tumor. Three months following the craniotomy, Gamma Knife radiotherapy was administered at a peripheral dose of 14 Gy, equating to a central dose of 31 Gy.

In December 2011, the patient developed bilateral hip pain and was diagnosed with femoral head necrosis, for which he was treated with traditional Chinese medicine. Endocrine investigations showed the level of plasma cortisol was 39.40 μg/L (normal range 67–226 μg/L) at 8 am, and the prednisone dose was decreased 2.5 mg once daily. In January 2013, he developed repeated episodes of lower limb edema, facial edema, and chemosis, and developed a round face, but did not seek treatment. In May 2013, the patient was admitted to his local hospital with an intracranial pyogenic infection. At that time he was noted to have hypocortisolemia and loss of circadian rhythms. The infection resolved with treatment, and the patient was advised to discontinue prednisone.

**FIGURE 3:**
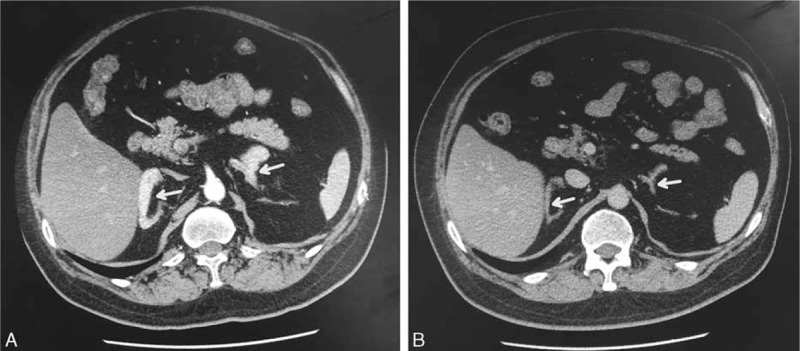
(A) Axial computed tomography (CT) images of the adrenals showing hyperplasia and enhancement of both glands before (white arrow) and (B) after the 4th surgical intervention. Both adrenal glands had almost returned to a normal size (white arrow).

In October 2014, the patient complained of worsening and repeated lower limb edema, facial edema, and chemosis. He was noted to have rapidly developed cushingoid facies, a buffalo hump, supraclavicular fat pads, central obesity, hypertension, hypokalemia, and osteoporosis. His BMI had increased substantially to 32.3 kg/m^2^. It was at this stage that the patient was referred to our hospital and came under our care. Further endocrine testing found elevated concentrations of ACTH and cortisol with loss of circadian rhythms, and substantially elevated 24-hour urinary-free cortisol (Table 1). A high-dose (8 mg) dexamethasone suppression test only slightly decreased serum cortisol concentration, from 2441.6 μg/24 h to 1524.64 μg/24 h. An overnight low-dose (1 mg) dexamethasone suppression test resulted in more than 50% suppression of cortisol concentration, which fell from 2441.6 μg/24 h to 780.08 μg/24 h. These equivocal results prompted a decision to perform inferior petrosal sinus sampling (IPSS) with administration of desmopressin acetate (DDAVP). The right inferior petrosal venous ACTH concentration increased following intravenous DDAVP administration, with a more than 3-fold increase in the ratio of the right inferior petrosal to peripheral concentration (Table 2). CT of the adrenals showed bilateral hyperplasia (Figure 3A), and a head CT scan revealed a space-occupying lesion in the sellar region, suggesting another recurrence of the pituitary adenoma (Figure 1B). Together, these findings provided unequivocal evidence of CD.

**TABLE 2:**
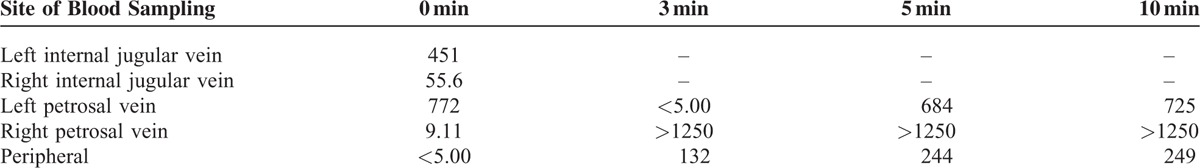
Adrenocorticotrophic Hormone Concentration (in pg/mL) During Inferior Petrosal Sinus Sampling/Desmopressin Testing

The patient was transferred to the neurosurgery department for another transsphenoidal resection of the pituitary adenoma. The histopathological examination was consistent with a pituitary adenoma (Figure 2D-F), but immunohistochemical staining for ACTH was positive (Figure 2E), and the Ki-67 proliferative index was 2% (Figure 2F). The postoperative course was uneventful, and the patient's fatigue and edema resolved. The concentrations of cortisol and ACTH remained elevated, with loss of circadian rhythms 7 days after surgery. Postoperative endocrine studies 3 months later found that the serum cortisol concentration was 90.0 ng/mL at 8 am, 58.2 ng/mL at 4 pm, and 75.1 ng/mL at midnight (normal range 50–250 ng/mL). The serum ACTH concentration was 124 pg/mL at 8 am, 102 pg/mL at 4 pm, and 109 pg/mL at midnight (normal range 0–46 pg/mL), and that the 24-hour urine-free cortisol remained elevated. A repeat abdominal CT scan showed that both adrenal glands were markedly smaller, close to the normal size (Figure 3B). Gamma Knife radiotherapy was recommended to control tumor growth and hypersecretion of ACTH.

## DISCUSSION

CD may be difficult to recognize, because the diagnostic techniques required are complex. Consequently, it is often misdiagnosed or diagnosed late in the disease process, meaning that endocrinologists and neurosurgeons have missed the opportunity to treat it promptly and effectively. Approximately 6% of all NFPAs are silent corticotroph adenomas (SCA),^18^ that is, pituitary adenomas with positive immunoreactivity for ACTH but without any signs or symptoms of Cushing syndrome; plasma ACTH concentrations are usually normal.^19^ In the setting of a lack of clinical or biochemical manifestations of hypercortisolism, differentiating between NFPAs and SCAs relies on histopathologic evidence of ACTH secretion.^20^

In our patient, imaging on presentation showed a pituitary macroadenoma with suprasellar extension, but he had no clinical features of hypercortisolism and a normal morning serum cortisol concentration. Unfortunately, serum ACTH concentration and 24-hour urinary-free cortisol were not measured at his first presentation. Cortisol concentration was, however, elevated before the second operation, which may have been a consequence of corticosteroid replacement therapy, and the possibility that there was intermittent CD with intermittent hypercortisolism cannot be entirely ruled out. Cyclical edema is reportedly found in about 15% of cases of CD, but its pathophysiology is not fully understood.^21^ It has been proposed that episodic hemorrhage and necrosis of cortisol-producing cells causes a cyclical release of cortisol, or that tumor calcification leads to periodic infarctions with subsequent eucortisolemia, but neither mechanism explains the periodicity. An alternative explanation is that ACTH hypersecretion is driven by pulsatile hypothalamic release of neurotransmitters including corticotropin-releasing hormone, norepinephrine, dopamine, acetylcholine, and γ-aminobutyric acid.^21^

Tissue obtained during excision of the second tumor contained some scattered ACTH-positive cells. Some pituitary adenomas exhibit an infiltrative growth pattern – occasionally trapping a core of nontumorous cells within the mass – resulting in partial positive immunohistochemical staining for hormones.^14^ This can lead to diagnostic confusion and has resulted in misdiagnoses of a “secreting adenoma.” In our patient, all 4 imaging studies were suggestive of a pituitary macroadenoma, while immunohistochemical findings suggested transformation from an NFPA to CD.

In this patient, the IPSS + DDAVP test indicated a central-peripheral ACTH ratio of greater than 3:1, which confirmed the diagnosis of CD. CD may be caused by hypersecretion of ACTH by the pituitary or one of the adrenals; if the latter is the focus of secretion, then the contralateral adrenal will atrophy.^22^ The finding of hyperplasia of both adrenals in our case implicated the pituitary tumor as the origin of hypersecretion, which is consistent with the finding that the adrenals had almost returned to their normal size 3 months following pituitary resection.

In 2007, the World Health Organization defined atypical pituitary adenomas as those with a Ki-67 expression >3%, excessive p53 expression and increased mitotic activity.^3^ Chiloiro et al^23^ have reported that pituitary adenomas with a Ki-67 expression ≥1.5% had a higher risk of recurrence and shorter disease-free survival compared with those with a Ki-67 expression <1.5%. In our patient, the Ki-67 expression between 1.5% and 3% reflected the tumor's aggressive clinical behavior resulting in repeated recurrences. Our findings also suggest that a Ki-67 expression ≥1.5% may be useful as a prognostic marker.

We undertook a retrospective analysis of the literature indexed in PubMed/Medline, EBSCO, Google Scholar, and the Wanfang and CNKI databases. There are 14 reports in the literature of secondary occurrence of CD from a tumor previously diagnosed as an SCA or NFPA.^4–17^ These cases share a variety of clinical features. All but one of the patients was women, and most were fertile when the tumor became active. The interval from the initial diagnosis of pituitary adenoma to recurrence ranged from months to 18 years, and most presented with visual field changes or menstrual disorders. The imaging findings revealed a macroadenoma in every case.

Female sex hormones tend to promote ACTH secretion, contributing to manifestations of CD. The difference between the sexes in the characteristics of pituitary gonadotropic adenomas first proposed by Horvath and Kovac^24^ has gained credence in the field of endocrinology. Vacuole-like changes in the Golgi apparatus may be an indication that an NFPA or female gonadotroph adenoma is changing its phenotype to an ACTH-secreting adenoma.^8,13^ The mechanism underlying these changes in the Golgi complex, and the reason why this change occurs only in females are not understood. The causes of the change in clinical phenotype from nonsecretory to ACTH secretion after Gamma Knife radiotherapy are also not clear, but a literature review pointed to several potential mechanisms. One possibility is that initially there was more than 1 tumor but the diagnosis of a single NFPA was made because, the related hormones were not produced in sufficient quantities to be detected. Loss of pituitary cell secretory function might also contribute to rapid growth.^25^

The hypothesis that gene mutations may result in various functional defects requires further evaluation. There may be other currently unrecognized genetic factors that may predispose tumors to phenotypic change in response to radiation. Although there have been no previous reports of radiotherapy-induced conversion of NFPAs to secretory tumors, there are reports suggesting that radiation may influence the clinical activity of pituitary tumors.^13,14^ The genes that may be susceptible to radiation and the homeostatic responses of cells to radiation injury are not known, and we have been unable to identify any factors that might predict which patients might develop CD. As our patient developed a corticotroph-secreting pituitary macroadenoma 3 years after Gamma Knife radiotherapy, we believe that the pituitary cell metamorphosis that caused CD might have been provoked by radiation.

In this patient, glucocorticoid therapy might have influenced the extent of excess ACTH secretion in the advanced stages of the disease. High-dose administration of exogenous hormone is likely to suppress secretion of endogenous hormones, disrupting negative feedback regulatory mechanisms, and potentially resulting in abnormal hormone concentrations.^26^ Beardwell et al^27^ have described a case of a male patient where glucocorticoid therapy contributed to ectopic ACTH secretion by a peripheral tumor, and Himsworth et al^28^ have reported the abnormal secretion of ACTH by a hepatic carcinoma.

Gel permeation chromatography can be used to detect high molecular weight ACTH, and immune electron microscopy can be used to observe the structure of the Golgi complex to rule out a silent ACTH adenoma in patients with an apparently nonfunctional macroadenoma. Clinically, patients with an ACTH-secreting adenoma generally have a normal or slightly elevated plasma cortisol concentration, but a substantially higher 24-hour urinary-free cortisol. Routine assessment of 24-hour urinary-free cortisol is therefore recommended for patients with an NFPA to assist in prompt diagnosis and treatment. Measurement of specific biomarkers, such as Ki-67, may help to inform risk prediction and personalized interventions for patients with pituitary ACTH-secreting adenomas.

The metabolic disorders brought about by CD cause clinical complications and increase mortality rate, and up to 25% of ACTH-secreting pituitary adenomas recur. Our patient is a man with an invasive ACTH macroadenoma with multiple recurrences who has experienced long-term subclinical CD – his prognosis is likely to be poor. This case stresses the importance of comprehensive, regular, and lifelong surveillance of patients with NFPAs, and close monitoring and control of serum cortisol concentration.
